# The epidemiology of silent brain infarction: a systematic review of population-based cohorts

**DOI:** 10.1186/s12916-014-0119-0

**Published:** 2014-07-09

**Authors:** Jonathon P Fanning, Andrew A Wong, John F Fraser

**Affiliations:** 1 School of Medicine, The University of Queensland, Brisbane, Queensland, Australia; 2 Critical Care Research Group (CCRG), The Prince Charles Hospital, Brisbane, Queensland, Australia; 3 Department of Neurology, The Royal Brisbane and Women’s Hospital, Brisbane, Queensland, Australia

**Keywords:** Silent brain infarcts, Cerebral infarction, Risk factors, Epidemiology

## Abstract

**Background:**

Cerebral infarction is a commonly observed radiological finding in the absence of corresponding, clinical symptomatology, the so-called silent brain infarction (SBI). SBIs are a relatively new consideration as improved imaging has facilitated recognition of their occurrence. However, the true incidence, prevalence and risk factors associated with SBI remain controversial.

**Methods:**

Systematic searches of the Medline and EMBASE databases from 1946 to December 2013 were performed to identify original studies of population-based adult cohorts derived from community surveys and routine health screening that reported the incidence and prevalence of magnetic resonance imaging (MRI)-determined SBI.

**Results:**

The prevalence of SBI ranges from 5% to 62% with most studies reported in the 10% to 20% range. Longitudinal studies suggest an annual incidence of between 2% and 4%. A strong association was seen to exist between epidemiological estimates of SBI and age of the population assessed. Hypertension, carotid stenosis, chronic kidney disease and metabolic syndrome all showed a strong association with SBI. Heart failure, coronary artery disease, hyperhomocysteinemia and obstructive sleep apnea are also likely of significance. However, any association between SBI and gender, ethnicity, tobacco or alcohol consumption, obesity, dyslipidemia, atrial fibrillation and diabetes mellitus remains unclear.

**Conclusions:**

SBI is a remarkably common phenomenon and endemic among older people. This systematic review supports the association of a number of traditional vascular risk factors, but also highlights disparities between clinically apparent and silent strokes, potentially suggesting important differences in pathophysiology and warranting further investigation.

## Background

Almost 50 years ago, Fisher [[1]] first described the presence of cerebral infarction in the absence of any clinically apparent stroke or transient ischemic attack. It is only in recent years with major advances in imaging technology, however, that ‘silent’ brain infarcts (SBI) have been studied in any detail. These lesions are not benign, as originally thought, and associations with subtle neurological deficits [[2],[3]], cognitive dysfunction [[4]–[6]], psychiatric disorders [[2],[7]–[9]], clinically apparent stroke [[10]–[13]] and early mortality [[4],[10]] have led to suggestions that the term ‘silent’ be replaced by ‘covert’ [[14]].

SBI is not a rare event, especially in the older population and certain other at-risk populations. However, the true incidence and prevalence of SBI remain controversial, and our understanding of risk factors limited. Current evidence is plagued by: large inter-study variance in epidemiological estimates, a striking disproportion of SBI research stemming from specific racial groups limiting generalizations to other populations, changing technologies that have influenced detection and even the SBI definition itself. Of the epidemiological literature available on SBI, the most credible and generalizable data come from large population-based cohort studies (for example, community samples and routine health screens).

This article presents robust incidence and prevalence estimates derived from general population studies identified by systematically reviewing the medical literature. Emphasis is placed not only on findings, but also on the strength of those findings, in terms of research methodology and consistencies between studies.

## Methods

According to the Preferred Reporting Items for Systematic Reviews and Meta-Analyses (PRISMA) statement, relevant papers were identified by means of electronic searches of abstracts published in English in the Medline and EMBASE databases between 1946 to December 2013 using advanced search functions and the search terms: ‘silent brain infarcts’, ‘silent cerebral infarcts’, ‘silent stroke’ and ‘silent lacunar infarcts’; these were combined with the terms: ‘epidemiology’, ‘incidence’, ‘prevalence’ or ‘risk factors’ [see Additional file 1: Table S1: Detailed Search Strategies]. As a second step towards detecting relevant studies, all references listed at the end of identified papers, or noted in footnotes or data tables, were reviewed. After removing duplicates, this process identified 745 records. Included studies were restricted to original contributions that prospectively enrolled adults from the population-based cohorts (via community surveys or routine health screening) and assessed for and specifically reported MRI-detected silent brain infarcts. To improve the reliability of derived conclusions, studies with sample sizes of <200 participants were excluded due to the large variability in incidence and prevalence estimates evident in smaller samples. A total of 64 articles covering 27 original studies were deemed eligible. In total, 33,671 patients and 36,582 MRI examinations were included and provided data for this review [see Additional file 2: Figure S1 for the PRISMA flow diagram]. Data were reviewed descriptively and collated in detailed tables (see Table 1 and Additional file 3: Table S2, Additional file 4: Table S3, Additional file 5: Table S4, Additional file 6: Table S5 to Additional file 7: Table S6). Where multiple articles were included from a single or overlapping population sample, prevalence and incidence estimates were obtained from the report with the largest sample size to prevent duplication.

**Table 1:** Prevalence and incidence of silent brain infarcts in population-based cohorts

**Study name or center**	**Authors**	**Year**	**Country**	**Study design**	**Sample size**	**Mean age**	**Mean prevalence**
**Prevalence**							
Seiryo Clinic Study	Asumi [[15]]	2010	Japan	RHS	324	54	5.2%
Shimane	Bokura [[10]]	2006	Japan	RHS	2,684	58	14%
	Bokura [[16]]	2010	Japan	RHS	1,543	62	14%
SNUH Gangnam MRI Survey	Kwon [[17]]	2006	Korea	RHS	1,588	54	5.5%
	Kwon [[18]]	2009	Korea	RHS	1,254	70	15.7%
Samsung Medical Centre	Lee [[19]]	2000	Korea	RHS	994	49	5.8%
‘Brain Dock’ program	Matsumoto [[20]]	2007	Japan	RHS	476	52	20.8%
Japanese elderly	Nakagawa [[21]]	2000	Japan	RHS	269	79	62%
Kawasaki medical school	Saji [[22]]	2012	Japan	RHS	220	69	25%
	Saji [[23]]	2012	Japan	RHS	240	69	17.5%
Hyogo Brain & Heart Centre	Uehara [[24]]	1999	Japan	RHS	219	63	40.2%
Second Military Medical University	Yi [[25]]	2011	China	RHS	1,008	49	32.5%
Chiba University	Yoshida [[26]]	2010	Japan	RHS	790	61	27.1%
KoGES	Cho [[27]]	2013	Korea	CS	746	59	7.6%
Kochi University	Park [[28]]	2008	Japan	CS	2,076	51	5.7%
Memorial Hospital Taipei	Chou [[29]]	2011	Taiwan	CS and RHS	1,312	52	5%
NILS-LSA	Kohara [[30]]	2003	Japan	CS	1,721	59	10.3%
FHOS	Das [[31]]	2008	USA	CS	2,040	62	11%
FHS	DeCarli [[32]]	2005	USA	CS	2,081	62	12%
ARICS	Howard [[33]]	1998	USA	CS	1,737	63	11%
ASPS	Schmidt R [[34]]	2006	Austria	CS	505	70	8%
Ohasama	Aono [[35]]	2007	Japan	CS	958	66	49%
NOMAS	Willey [[36]]	2011	USA	CS	1,238	70	16%
MEMO	Schmidt WP [[37]]	2004	Germany	CS	267	72	12.7%
Rotterdam Scan	Vermeer [[38]]	2002	Netherlands	CS	1,077	72	20%
CHS*	Price [[3]]	1997	USA	CS	3,397	75	28%
Sefuri Brain MRI	Fukuda [[39]]	2013	Japan	CS	715	67	12.4%
3C-Dijon	Satizabal [[40]]	2012	France	CS	1,841	72.5	8.9%
TASCOG	Choi [[41]]	2012	Australia	CS	351	72	12.2%
CABL	Russo [[42]]	2013	USA	CS	455	70	15.4%
**Study name or center**	**Authors**	**Year**	**Country**	**Study design**	**Sample size**	**Mean age**	**Mean incidence**
**Incidence**							
ARICS	Cheung [[43]]	2010	USA	CS	810	62	1.9%
CHS*	Longstreth [[14]]	2002	USA	CS	1,433	74	3.1%
Rotterdam Scan Study	Vermeer [[44]]	2003	Netherlands	CS	668	71	3.7%

*study reported silent lacunar (subcortical) infarcts only. ARICS, Atherosclerosis Risk in Communities Study; ASPS, Austrian Stroke Prevention Study; CABL, Cardiovascular Abnormalities and Brain Lesions; CHS, Cardiovascular Health Study; CS, community sample; FHOS, Framingham Heart Offspring Study; FHS, Framingham Heart Study; KoGES, Korean Genome and Epidemiology Study; MEMO, Memory and Morbidity in Augsberg Elderly; NILS-LSA, National Institute for Longevity Sciences-Longtitudinal Study of Aging; NOMAS, Northern Manhattan Study; RHS, routine health screen; SNUH, Seoul National University Hospital; TASCOG, Tasmanian Study of Cognition and Gait.

## Results and discussion

### Incidence and prevalence of SBI

**Figure 1:**
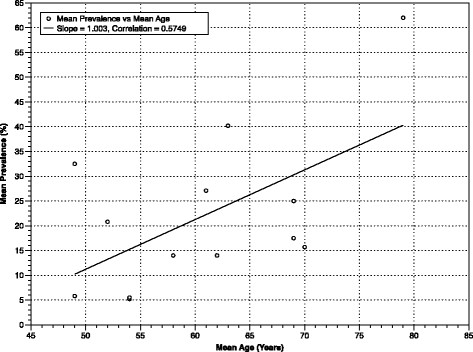
Mean Prevalence of SBI, by Mean Age in Clinic Patients Undergoing Routine Health Screening.

**Figure 2:**
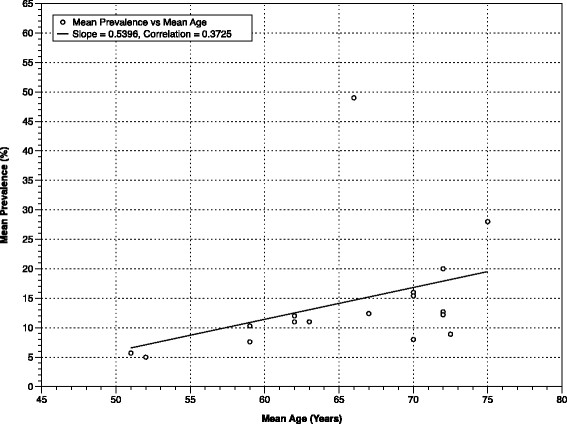
Mean Prevalence of SBI, by Mean Age in General Community Surveys.

**Figure 3:**
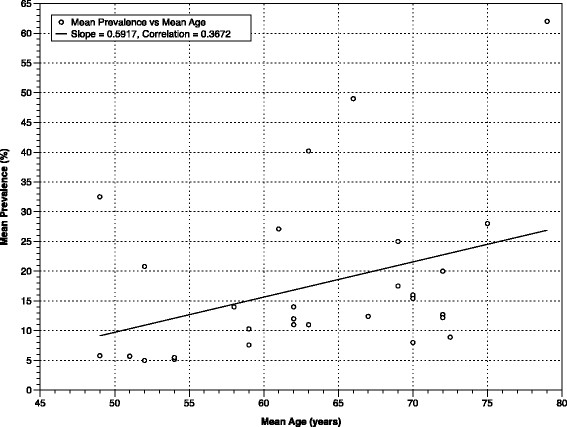
**Mean Prevalence of SBI, by Mean Age in population-based cohorts (combining Figures**1**and**2**).**

Studies on the incidence and prevalence of SBI in population-based cohorts have primarily been conducted in two settings: representative community samples (CS) and participants undergoing routine health screening (RHS). Community samples have generated SBI prevalence estimates among non-institutionalized, asymptomatic participants from as low as 5.7% in 746 individuals between the ages of 50 and 79 living in Korea [[27]], to as high as 49% in 958 volunteers 55-years old and older in Ohasama, Japan [[35]]. Most remaining published CS studies give estimates in the 10% to 20% range (Table 1). In those undergoing RHS, the range has been greater still, from 5% [[29]] to 62% [[21]]. It is clear from these data that a statistically significant correlation exists between mean SBI prevalence and mean sample age, both when all 26 studies providing SBI prevalence data are compiled, and when community and clinic studies are assessed separately (Figures 1, 2 and 3). This same age-dependent pattern is evident by region when Asian, American, and European studies are assessed separately [see Additional file 8: Figure S2].

The inter-study association concerning mean subject age and SBI prevalence is consistent with intra-study data from a number of sources. In the landmark Rotterdam Scan Study, Vermeer *et al*. studied 1,077 community residents between 60- and 90-years old, among whom the prevalence of SBI rose steadily, from 8% in those 60- to 64-years old and 13% in those 65- to 69-years old, to more than 20% in the 70 to 79 age group, and 35% in those older than 80 [[38]]. The adjusted odds ratio (OR) for age was 1.08 per year (95% confidence interval (CI): 1.05, 1.10), indicating an average 8% increase in the odds of having a SBI for each year lived over age 60. Similarly, in another survey of 994 neurologically-healthy adults between the ages of 20 and 78 years in Seoul, Lee *et al.* identified no SBI in individuals 20- to 39-years old, with SBI prevalence rising steadily with every subsequent decade to 1.7% in 40- to 49-year olds, 9.2% in 50- to 59-year olds, 19.8% in 60- to 69-year olds and 43.8% in 70- to 79-year olds (*P* <0.001) [[19]]. In this survey, the odds of having a new SBI increased by 13% with each year lived (OR: 1.13; 95% CI: 1.09, 1.18). Also in Seoul, Kwon *et al.* twice found a significant increase in SBI prevalence with age: once in a survey of 1,254 individuals undergoing RHS [[18]], with lesions noted in 11.7% of the 65- to 69-year-old sample, in 18.0% of those 70- to 74-years old, and in 27.6% of those older than 75; and among 1,588 neurologically-healthy subjects who underwent brain MRI as part of a RHS [[17]], the prevalence of lesions ranging from 1.4% in those 40-years old and younger to 17.5% in those 70-years old and older. Clearly then, although the prevalence of SBI varies widely across general populations in the literature, there is a strong association with age, and prevalence rates approaching and even surpassing 20% are not uncommon in those older than 70.

The prevalence of SBI in Asian countries appears to be greater than in non-Asian countries (20.2% in Asia, 12.4% in Europe and 15.6% in the USA, *P* = 0.017; Additional file 8: Figure S2a). Moreover, this effect is independent of age, as Asian samples were the youngest (mean age = 59.5, 71.6 and 67.0 years, respectively). However, this apparent increased prevalence in Asian countries is likely an artifact, caused by 11 of the 17 Asian studies being RHS, in which the prevalence of SBI tended to be greater (mean prevalence 23.1% versus 15.0% in RHS and CS, respectively), while every non-Asian study was a community survey. When community surveys alone are examined, any apparent increase in SBI prevalence in Asia is corrected [see Additional file 8: Figure S2b], and the mean per-study prevalence rate estimates for Asian (n = 6), European (n = 4), American (n = 6) and Australian studies become equivalent (17.0, 12.4, 15.6 and 12.2%, respectively). Moreover, when the one clear outlier study (Aono *et al*., [[35]]; prevalence 49%) is excluded, the mean prevalence estimate for the five remaining Asian studies falls to 9.0%, the lowest mean prevalence. Importantly, the average age across the remaining Asian community surveys was younger, again supporting mean sample age as the primary determinant of prevalence in different community samples.

Due to the inherent difficulties of longitudinal studies, incidence data are scarce, with only three identified studies providing estimates that range from 1.9% to 3.7% per year [[14],[43],[44]]. Once again, age was a predictor of incidence, with 8% of those 60- to 69-years old having new lesions over the duration of follow-up versus 22% of those 80 and older [[24]]. Thus, the association between age and SBI prevalence reflects both increased incidence with age and the cumulative effect of time.

Contrary to what might be expected due to advancing technologies and perhaps enhanced detection of SBI, there has been no apparent rise in SBI prevalence over the years. In fact, from 2010 onwards, the highest SBI prevalence rate reported in a CS has been 16% [[36]]. Neither can we speculate that prevalence is decreasing, despite apparently falling rates, because of the clear shift in study design from RHS to randomly recruited CS samples, the latter accounting for seven of the eight studies conducted since 2010.

### Risk factors

It is from community-based samples, examined using logistic regression that the best understanding emerges about the prevalence of SBI in various potentially at-risk populations. Table 2 presents a summary of the risk factors examined and the strength of their association with SBI.

**Table 2:** Summary of strength of association of risk factors with silent brain infarction

**Strength of association**	**Risk factors**
Strong	Age
	Hypertension
	Metabolic syndrome
	Carotid artery disease
	Chronic kidney disease
Likely	Coronary artery disease
	Heart failure
	Homocysteinemia
	Obstructive sleep apnea
Unclear	Atrial fibrillation
	Dyslipidemia
	Diabetes
	Obesity
	Alcohol
	Tobacco
	Ethnicity
	Gender

### Demographic factors

#### Age

Consistent with the described associations between sample age and the reported prevalence/incidence of SBI, age has been one of the most clearly identified risk factors for SBI. In fact, of the 19 included studies in which an association between age and SBI rates was sought, such an association was identified in 18 [see Additional file 3: Table S2], with the OR of prevalent SBI assessed per year of age ranging from 1.03 (95% CI: 0.98, 1.08) [[23]] to 1.13 (95% CI: 1.09, 1.18) [[19]] and per decade ranging from 2.44 (95% CI: 1.84, 3.23) [[39]] to 3.21 (95% CI: 2.17, 4.74) [[33]]. In contrast to age, studies have failed to convincingly identify significant risk associated with any other demographic characteristic.

#### Gender

Vermeer *et al*. [[38]] identified an odds ratio for SBI prevalence of 1.4 (95% CI: 1.0, 1.8) associated with female gender in the Rotterdam Scan Study; but this OR became non-significant (OR: 1.3; 95% CI: 0.9, 1.9) when adjusted for patient age, hypertension, diabetes and smoking. A similar OR of 1.4 was found for female gender when SBI incidence was examined; but this too failed to be statistically significant (95% CI: 0.6, 3.2) [[44]]. Conversely, Longstreth *et al.* [[45]] discovered male gender to be protective (OR: 0.74; *P* <0.005) against lacunar infarcts in an MRI study of 3,660 older adults (≥65 years of age). The majority of studies, however, do not support any gender disparity in SBI risk [[3],[14],[30],[32],[33],[35],[37],[44],[46]]. Among women, it is interesting to note recent evidence that links early menopause with a four-fold increased risk of SBI [[39]].

#### Ethnicity

To date, few data support any association between SBI and race, contrary to the significantly increased risk of stroke and related mortality and morbidity identified among African Americans [[47]]. This may be due to confounding factors including hypertension, diabetes and obesity [[47]]. One notable exception among identified SBI studies is the USA-based Atherosclerosis Risk in Communities (ARIC) Study [[33]] in which, among 1,737 participants selected from the general population, multiple variable analysis identified ‘non-white’ status as significantly associated with an increased odds of SBI (OR: 1.64; 95% CI: 1.12, 2.41) [[33]]. One limitation to current research evaluating race as a risk factor for SBI is that a disproportionate number of SBI studies were conducted in ethnically homogeneous populations, notably in Japan and Korea.

### Lifestyle factors

#### Tobacco

In only three [[33],[39],[48]] out of 15 studies in which an association was sought has tobacco been identified as a statistically significant risk factor for SBI [see Additional file 4: Table S3]. Of particular note is that, although the Rotterdam Scan Study identified a statistically significant increase in clinically apparent stroke (OR: 3.1; 95% CI: 0.6, 1.5), no such association was demonstrated for SBI even among the heaviest smokers with ≥20 pack year histories (OR: 1.0; 95% CI: 0.6, 1.5) [[38]].

#### Alcohol

It is not surprising — given reports of the protective role of modest daily alcohol consumption, particularly red wine, against cardiovascular disease [[49]] — that an association was sought with SBI in eight of the studies we reviewed [see Additional file 4: Table S3]. A protective role was identified by Lee *et al*., [[19]] for mild (one to two times per week) alcohol consumption (OR: 0.31; 95% CI: 0.12, 0.78) and by Mukamal *et al*., [[50]] for between one and six standard drinks per week (OR: 0.63; 95% CI: 0.46, 0.86). Interestingly, the latter study also suggested a protective role of heavy alcohol intake with odds of SBI associated with consumption of ≥15 standard drinks per week of 0.57 (95% CI: 0.32, 1.0) [[50]]. However, results have been inconsistent, with other studies [[39],[48],[51]] identifying an increased risk of SBI with alcohol consumption to as high as OR: 4.1 (95% CI: 1.7,10) [[51]]. No effect, protective or otherwise, was seen comparing those who have never consumed alcohol against those who have [[35]], or when looking at past [[33],[50]] or minimal alcohol consumption (<1 standard drink per week) [[50]]. Ethnic differences in alcohol metabolism likely confound our understanding of the true effect of alcohol. For example, every study that identified an increased risk of SBI associated with alcohol consumption was conducted in Japan.

### Cardiovascular risk factors

#### Obesity

Conflicting results are noted for obesity [see Additional file 5: Table S4]. In the largest study, Park *et al*. [[28]] identified an increased risk of SBI with waist circumference ≥102 cm (male) or ≥88 cm (female) (highest versus lowest tertile, OR: 4.3; 95% CI: 2.4, 7.71 and dichotomized OR: 8.35; 95% CI: 5.59, 12.5). Bokura *et al.*, [[52]] also identified increased risk with a BMI ≥25 kg/m^2^ (OR: 1.55; 95% CI: 1.05, 2.27). However, others have not corroborated these results. In fact, a number of investigators [[17],[33],[35]] have identified protective effects, albeit only statistically so in one of three studies (OR: 0.72 for BMI ≥25 versus <25 kg/m^2^; 95% CI: 0.53, 0.97) [[35]].

#### Dyslipidemia

ORs for measures of dyslipidemia (total cholesterol, high density lipoproteins, low density lipoproteins and triglycerides) have varied widely [see Additional file 6: Table S5]. Only four of 25 estimates across 15 studies have demonstrated a significantly positive association with SBI rate, that being single OR estimates for serum triglycerides (OR: 2.82; 95% CI: 1.83, 4.33) [[28]], total cholesterol per mmol/L (OR: 3.75; 95% CI: 1.45, 9.68) [[15]], LDL per mmol/L (OR: 2.54; 95% CI: 1.03, 6.27) [[15]] and HDL per standard deviation (OR:1.13; 95% CI: 1.03, 1.23) [[25]].

#### Homocysteinemia

More consistent are the results for total plasma homocysteine, a documented risk factor for clinical stroke [[53],[54]]. As demonstrated in Additional file 6: Table S5, three [[31],[55],[56]] of six ORs were statistically greater than 1.00; and a fourth, in which SBI incidence rather than prevalence was studied, just failed to achieve statistical significance (OR: 1.31; 95% CI: 0.95, 1.82) [[44]]. In the Framingham Offspring Study [[56]], not only was an association between plasma homocysteine levels and SBI rates noted, but also the strength of this association increased with age. Further supporting a role for homocysteine, MTHFR C677T genetic polymorphism is independently associated with almost double (OR: 1.72; 95% CI: 1.10, 2.68) the odds of SBI versus more common haplotypes, where normally functioning MTHFR metabolizes homocysteine to methionine [[30]].

### Cardiovascular disease states

#### Hypertension

Hypertension is the cardiovascular risk factor for which the strongest association with risk of SBI has been identified, and it consistently ranks as one of the top two risk factors overall. In fact, all 20 studies designed to identify an association between SBI and hypertension have detected one, although this was only statistically significant in 18 [see Additional file 7: Table S6]. The highest odds were reported by Fukuda *et al*., [[39]] of OR 4.04 (95% CI: 2.41, 6.77).

#### Carotid and coronary artery disease

Diseases of the carotid and coronary arteries comprise significant risk factors for prevalent SBI, with odds ratios consistently greater than one (Additional file 7: Table S6). Although carotid disease has been variably evaluated, all but one of the studies we reviewed identified a statistically significant association between at least one such measure and SBI [[31],[44],[45],[57]], with ORs as high as 5.51 (95% CI: 1.31, 23.1) [[15]]. Likewise, a statistically significant association was identified in four of seven analyses of coronary artery disease, with odds ratios up to 2.83 (95% CI: 1.38, 5.82) [[17]].

#### Atrial fibrillation and heart failure

Despite the obvious implications of atrial fibrillation for thromboembolism, the Framingham Offspring Study [[31]] was the only one of three community-based studies to identify a statistically significant association with SBI [see Additional file 7: Table S6]. Here, atrial fibrillation was the disease with the highest odds of an individual having had at least one SBI (OR: 2.16; 95% CI: 1.07, 4.40), followed by hypertension (OR: 1.56; 95% CI: 1.15, 2.11) [[31]]. Interestingly, despite these findings, the ORs for combined cardiovascular disease lacked statistical significance (OR: 1.38; 95% CI: 0.87, 2.18) [[31]].

The association between prevalent SBI and measures of heart failure has been more consistent [see Additional file 7: Table S6]. The landmark population study in this regard is the cardiovascular abnormalities and brain lesion (CABL) study [[42]], which identified significantly increased odds of SBI in those with a dilated left atrium, as determined by minimum volume (OR: 1.37; 95% CI: 1.04, 1.80) and ejection fraction (OR: 1.49; 95% CI: 1.11, 2.00), independent of traditional cardiovascular risk factors.

### Non-cardiovascular diseases

#### Diabetes mellitus and chronic kidney disease

Surprisingly, despite the well-established role of diabetes mellitus as a vascular risk factor, logistic regression analysis of eligible studies suggests that its role in SBI may be less than anticipated [see Additional file 5: Table S4]. Seventeen community-based surveys have assessed for measures of impaired glycemic control, with ORs reported from 0.38 (95% CI: 0.05, 2.60) [[15]] to as high as 3.26 (95% CI: 1.09, 9.77) [[39]], seven of which identified a statistically significant increased risk. An eighth study, the longitudinal Rotterdam Scan Study [[44]], just failed to achieve statistical significance (OR: 2.9; 95% CI: 1.00, 8.5).

Evidence is stronger for an association between SBI and chronic kidney disease, with odds as high as 10.56 (95% CI: 3.00, 37.10; Additional file 5: Table S4). In fact, of the studies examined in this review, four of five demonstrate a statistically significance relationship between prevalent SBI and levels of serum creatinine [[45]], estimated glomerular filtration rate [[29]] or cystatin C [[58]]. An association between SBI incidence and serum creatinine (OR: 1.5; 95% CI: 1.00, 2.40) [[14]] was also suggested.

Both diabetes and chronic kidney disease patients suffer multiple cardiovascular co-morbidities, for which associations with SBI have been described. Consequently, whether these co-morbidities or the diabetes or kidney disease, *per se*, are responsible for the positive relationship with SBI remains speculative.

#### Metabolic syndrome

Three studies [[17],[28],[52]] have specifically assessed for an association between SBI and so-called metabolic syndrome, which incorporates hypertension, impaired fasting glucose, elevated serum triglyceride levels, low levels of high density lipoproteins and obesity [see Additional file 5: Table S4]. Predictably, given the clear association documented between SBI and hypertension, the odds of SBI were increased in each study. Whether this reflects anything more than the association of metabolic syndrome with hypertension warrants further research.

#### Other potential associations

Other reported associations with SBI rates [see Additional file 6: Table S5] include hyperuricemia [[59]], fibrinogen [[35],[60]] and, with a protective effect, the anticoagulant factor Protein C [[60]]. Cho *et* al. [[27]] also demonstrated that moderate-to-severe obstructive sleep apnea also significantly increases risk in those older than 65 (OR: 2.44; 95% CI: 1.31, 9.23).

### Limitations

Modern detection of SBI is dependent upon the sensitivity and specificity of imaging and definition of the radiological appearance. However, these have been major sources of heterogeneity in the literature with important implications for SBI incidence and prevalence estimates [[61]]. MRI parameters have varied widely between studies with magnet strength ranging from 0.02 to 1.5 Tesla (T) and section thickness from 4 mm to greater than 6 mm altering MRI sensitivity for SBI detection between studies. With regard to defining SBI, inconsistent radiological criteria for diagnosing infarction have been applied. Consequently, prevalence is underestimated by definitions limiting SBIs to lacunes or lesions with cerebrospinal fluid (CSF)-like signal intensity (reflecting only completed and cavitated infarcts) in light of evidence that 30% to 80% of lacunes do not cavitate [[62],[63]]. Conversely, more inclusive signal definitions, including lesions ≤3 mm in diameter overestimate prevalence. A common source of misdiagnosis are dilated perivascular spaces (dPVS), also known as Virchow-Robin spaces, although they tend to be smaller, generally less than the 3 mm minimum diameter set as a threshold for most SBI studies, and more commonly ovoid or linear in a periventricular orientation, versus irregularly-margined, round to wedge-shaped, and having a hyper-intense rim on fluid attenuated inversion recovery (FLAIR) sequences. Despite these differences, comparisons between imaging and autopsy findings have found that dPVS are misdiagnosed as SBI 10% to 20% of the time [[64]].

These differing definitions are likely to have important implications for understanding as different risk factors and prognostic profiles may exist for patients with different subtypes (cortical versus lacunar) and numbers of SBI (single versus multiple) [[45]]. This is particularly apparent in studies that consider any cerebral infarction without clinical symptomatology as silent brain infarction, thus equating small vessel disease lacunar infarcts with larger vessel cortical infarcts presumed secondary to arteriosclerosis or embolic phenomenon. For the current review, only the broad concept of silent versus clinically apparent infarction was assessed.

Pervading the literature on SBI is the inherent issue of selection bias, which can be seen to manifest in a number of forms. Classification of infarction as silent often relies on patient recollection of symptoms consistent with stroke. The validity of such self-report methods remains inconclusive [[65]], and high prevalence of stroke symptoms have been reported among persons without a diagnosis of stroke [[66]]. Additionally, to ensure that no clinically apparent stroke is mistaken as silent infarction, this analysis excluded participants who reported a history of stroke. However, silent strokes are a major risk factor for the development of a clinically apparent stroke, hence, a large portion of stroke patients will have additional silent infarctions, all of which have not been considered in this analysis.

Publication bias is also inherent in any systematic literature review and may have resulted in a biased sample of all relevant studies on the topic. Specifically, it is well established that studies reporting relatively large effects are more likely to be published than those that do not, and this becomes more pertinent among studies with smaller sample sizes [[67]]. Publication bias, along with heterogeneity in methodological quality, are two possible mechanisms explaining the pattern of larger effect sizes in small published studies. As explained in the Methods section, this ‘small-study effect’ was the rationale for excluding studies with <200 participants.

Finally, reporting across the SBI literature has consistently relied on multivariable models to determine individual risk associated with a variable. However, the extent to which vascular risk factors are independent has been questioned [[68]] and understanding of interactions between risk factors is largely lost by such analytic methods.

### Future directions

The abovementioned limitations provide a number of key considerations for potential studies examining this important area. In particular, the application of consistent detection criteria is crucial and any attempts to further investigate SBI epidemiology should take note of the criteria upon which recent studies have converged. As a minimum, MRI field strength of 1.5 tesla should be employed and lesions of less than 3 mm in size excluded to minimize misdiagnosis of dilated perivascular spaces.

The relevance of a comprehensive understanding of SBI epidemiology is highlighted by the increasingly appreciated morbidity and mortality associated with their occurrence and, thus, the potential to identify patients at risk of these poor outcomes. Furthermore, appreciation for SBI in the general population is essential to understand its implications in specific disease populations and procedures – a setting where SBI is gaining increasing acceptance as a surrogate marker for brain injury [[69]] and primary endpoint for related research [[70]].

## Conclusions

Due to the absence of overt clinical impairment, silent brain infarction is an under-investigated and poorly understood entity. Comprehension is further obscured by significant heterogeneity in diagnostic strategy for SBI detection which limits the comparability of studies. The prevalence of SBI ranges from 5% to 62% in population-based cohorts, most estimates falling in the 10% to 20% range. Longitudinal studies suggest an annual incidence between 2% and 4%.

Although clear associations exist, it would be overly simplistic to assume that entirely the same mechanisms underlie all types of cerebrovascular event. Much more is known about risk factors for SBIs than the pathogenic mechanisms behind them. As such, understanding the former provides a useful platform for directed investigation of the latter.

Age and hypertension are the two factors associated with the strongest risk of SBI. Overall cardiovascular health (including coronary artery disease, homocysteinemia, carotid artery stenosis and previous cerebrovascular events), diabetes, renal impairment and metabolic syndrome are also likely of significance, although the data are less consistent. The effect of alcohol appears to depend on the population studied, with mild-to-moderate alcohol consumption appearing protective except among Japanese populations, where it increases the risk of SBI. Surprisingly, no consistent associations have yet been identified between SBI rates and smoking, gender, ethnicity, dyslipidemia or obesity.

## Competing interests

The authors declare that they have no competing interests.

## Authors’ contributions

JPF was responsible for the conception and design of study, analysis and interpretation of data and manuscript preparation. AAW and JFF were involved in the interpretation of data and critically revising the manuscript for important intellectual content. All authors read and approved the final manuscript.

## Additional files

## Supplementary Material

Additional file 1: Table S1. Detailed search strategies. Click here for file

Additional file 2: Figure S1. PRISMA Flow Diagram. Click here for file

Additional file 3: Table S2. Age as a risk factor for Silent Brain Infarct Prevalence. Click here for file

Additional file 4: Table S3. Substance use as a risk factor for prevalent Silent Brain Infarct. Click here for file

Additional file 5: Table S4. Non-cardiovascular Disease risk factors for prevalent Silent Brain Infarction. Click here for file

Additional file 6: Table S5. Laboratory indicators as risk indicators of prevalent Silent Brain Infarction. Click here for file

Additional file 7: Table S6. Cardiovascular disease as a risk factor for prevalent Silent Brain Infarct. Click here for file

Additional file 8: Figure S2a. Mean prevalence of silent brain infarction, by mean age, differentiated by origin of study. Click here for file
