# Supplementary material for: The epidemiology of silent brain infarction: a systematic review of population-based cohorts
Source: BMC Med. 2014 Jul 9;12:119. doi: 10.1186/s12916-014-0119-0 (PMC4226994; doi:10.1186/s12916-014-0119-0)
Supplement: Additional file 1: Table S1. — Detailed search strategies. [file s12916-014-0119-0-S1.docx]

**Supplementary Table 1:** Detailed search strategies

| A. Database: Ovid MEDLINE(R), Ovid MEDLINE(R) In-Process & Other Non-Indexed Citations, Ovid MEDLINE(R) Daily and Ovid OLDMEDLINE(R) <1946 to Present> | |
| --- | --- |
| 1. | ep.xs. (1613392) |
| 2. | (prevalence or incidence).ti,ab,hw. (1003025) |
| 3. | 1 or 2 (2114479) |
| 4. | ((brain infarct* or cerebral infarct* or stroke*) adj3 silent).ti,ab. (630) |
| 5. | exp Stroke/ (85762) |
| 6. | silent.ti,ab. (30897) |
| 7. | 5 and 6 (883) |
| 8. | 4 or 7 (1057) |
| 9. | 3 and 8 (502) |
| 10. | limit 9 to english language (463) |
| 11. | risk factors/ (576119) |
| 12. | 8 and 11 (387) |
| 13. | (silent atrial or silent AF).ti,ab. (43) |
| 14. | 12 not 13 (382) |
| 15. | limit 14 to english language (340) |
| 16. | 10 or 15 (553) |
| B. Database: Embase <1980 to 2013 Week 48> | |
| 1. | exp *cerebrovascular accident/ (30154) |
| 2. | silent.ti,ab. (34724) |
| 3. | 1 and 2 (201) |
| 4. | ((brain infarct* or cerebral infarct* or stroke*) adj3 silent).ti,ab. (865) |
| 5. | 3 or 4 (987) |
| 6. | ep.fs. (938626) |
| 7. | exp epidemiology/ (1903300) |
| 8. | risk factor/ (611920) |
| 9. | or/6-8 (2704492) |
| 10. | 5 and 9 (553) |
| 11. | limit 10 to english language (504) |
