# Supplementary figures and images for: The epidemiology of silent brain infarction: a systematic review of population-based cohorts
Source: BMC Med. 2014 Jul 9;12:119. doi: 10.1186/s12916-014-0119-0 (PMC4226994; doi:10.1186/s12916-014-0119-0)

## PRISMA Flow Diagram

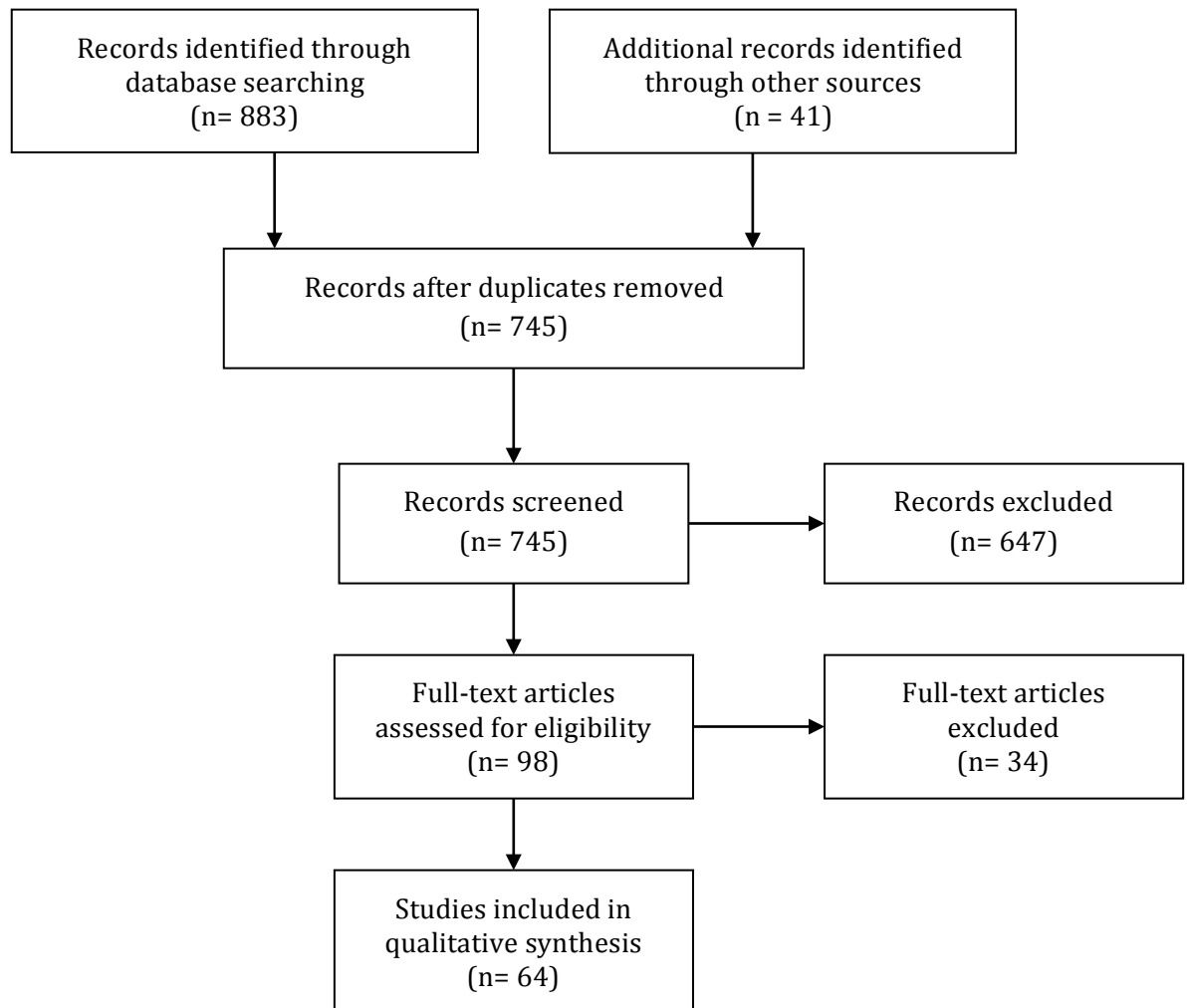

Supplement: Additional file 2: Figure S1. — PRISMA Flow Diagram. [file s12916-014-0119-0-S2.pdf]
