# Supplementary material for: The epidemiology of silent brain infarction: a systematic review of population-based cohorts
Source: BMC Med. 2014 Jul 9;12:119. doi: 10.1186/s12916-014-0119-0 (PMC4226994; doi:10.1186/s12916-014-0119-0)
Supplement: Additional file 3: Table S2. — Age as a risk factor for Silent Brain Infarct Prevalence. [file s12916-014-0119-0-S3.docx]

**Supplementary Table 2:** Age as a risk factor for Silent Brain Infarct Prevalence

| Study | Year | Country | Design | Condition | Size | OR | 95% CI |
| --- | --- | --- | --- | --- | --- | --- | --- |
| Aono^[1](#_ENREF_1)^ | 2007 | Japan | CS | Age (per decade) | 958 | 2.90 | 2.22 -3.80 |
| Asumi^[2](#_ENREF_2)^ | 2010 | Japan | RHS | Age (per year) | 324 | 1.12 | 1.03 – 1.21 |
| Bokura^[3](#_ENREF_3)^ | 2008 | Japan | RHS | Age (per year) | 1151 | 1.07 | 1.04 - 1.10 |
| Fukuda[^4^](#_ENREF_4) | 2013 | Japan | CS | Age (per decade) | 715 | 2.44 | 1.84 – 3.23 |
| Heo^[5](#_ENREF_5)^ | 2010 | Korea | RHS | Age (per year) | 1577 | 1.05 | 1.02 – 1.08 |
| Howard[^6^](#_ENREF_6) | 2000 | USA | CS | Age (per decade) | 1737 | 3.21 | 2.17 – 4.74 |
| Kwon[^7^](#_ENREF_7) | 2006 | Korea | RHS | Age (per year) | 1588 | 1.06 | 1.04 – 1.09 |
| Kwon[^8^](#_ENREF_8) | 2009 | Korea | RHS | Age (per year) | 1254 | 1.09 | 1.05 – 1.12 |
| Lee[^9^](#_ENREF_9) | 2000 | Korea | RHS | Age (per year) | 994 | 1.13 | 1.09 – 1.18 |
| Longstreth^[10](#_ENREF_10)^ | 1998 | USA | CS^Φ^ | Age ≥ 76 vs. ≤ 68 | 3660 | 1.66 | 1.27 – 2.16 |
| Longstreth^[11](#_ENREF_11)^ | 2002 | USA | CS* | Age >75 vs. <65 | 1433 | 1.5 | 1.0 – 2.4 |
| Matsumoto[^12^](#_ENREF_12) | 2007 | Japan | RHS | Age (per year) | 476 | 1.12 | 1.08 – 1.17 |
| Price[^13^](#_ENREF_13) | 1997 | USA | CS | Age (per quartile) | 3647 | 1.37 | 1.22 – 1.55 |
| Saji^[14](#_ENREF_14)^ | 2012 | Japan | RHS | Age (per year) | 220 | 1.07 | 1.03 – 1.12 |
| Saji^[15](#_ENREF_15)^ | 2012 | Japan | RHS | Age (per year) | 240 | 1.03 | 0.98 – 1.08 |
| Takashima[^16^](#_ENREF_16) | 2010 | Japan | CS | Age (per decade) | 680 | 2.76 | 2.04 – 3.74 |
| Vermeer[^17^](#_ENREF_17) | 2002 | Netherlands | CS | Age (per year) | 1077 | 1.08 | 1.05 – 1.10 |
| Vermeer[^18^](#_ENREF_18) | 2003 | Netherlands | CS* | Age (per year) | 668 | 1.08 | 1.04 – 1.13 |
| Yi[^19^](#_ENREF_19) | 2011 | China | RHS | Age (per year) | 1008 | 1.09 | 1.07 – 1.11 |

^Φ^ Silent lacunes only assessed (i.e. excludes silent cortical infarcts); * longtitudinal study association with SBI incidence; RHS = Routine Health Screen; CS = Community Survey
