# Supplementary material for: The epidemiology of silent brain infarction: a systematic review of population-based cohorts
Source: BMC Med. 2014 Jul 9;12:119. doi: 10.1186/s12916-014-0119-0 (PMC4226994; doi:10.1186/s12916-014-0119-0)
Supplement: Additional file 4: Table S3. — Substance use as a risk factor for prevalent Silent Brain Infarct. [file s12916-014-0119-0-S4.docx]

**Supplementary Table 3:** Substance use as a risk factor for prevalent Silent Brain Infarct

| **Study** | **Year** | **Country** | **Design** | **Measure** | **Size** | **OR** | **95% CI** |
| --- | --- | --- | --- | --- | --- | --- | --- |
| **CIGARETTES** | | | | | | | |
| Aono^[1](#_ENREF_1)^ | 2007 | Japan | CS | Ever smoker | 958 | 1.19 | 0.74 – 1.90 |
| Asumi^[2](#_ENREF_2)^ | 2010 | Japan | RHS | Current smoker vs. lifelong non-smoker | 324 | 2.6 | 0.76 – 8.91 |
| Bokura^[3](#_ENREF_3)^ | 2008 | Japan | RHS | Non-smoker | 1151 | 0.87 | 0.55-1.34 |
| Das[^4^](#_ENREF_4) | 2008 | USA | CS | Current smoker | 2040 | 1.34 | 0.90 – 1.99 |
| Fukuda[^5^](#_ENREF_5) | 2013 | Japan | CS | ≥10 cigarettes / day | 715 | 2.95 | 1.52 – 5.69 |
| Heo^[6](#_ENREF_6)^ | 2010 | Korea | RHS | Smoking history | 1577 | 1.20 | 0.72 – 1.99 |
| Howard[^7^](#_ENREF_7) | 1998 | USA | CS | Current smoker | 1737 | 1.88 | 1.13 – 3.13 |
| Howard[^7^](#_ENREF_7) | 1998 | USA | CS | Past smoker | 1737 | 1.16 | 0.74 – 1.83 |
| Howard[^7^](#_ENREF_7) | 1998 | USA | CS | Environmental smoke | 1737 | 1.06 | 0.64 – 1.75 |
| Lee[^8^](#_ENREF_8) | 2000 | Korea | RHS | Current smoker | 994 | 2.08 | 0.97 – 4.47 |
| Lee[^8^](#_ENREF_8) | 2000 | Korea | RHS | Past smoker | 994 | 0.45 | 0.09 – 2.26 |
| Longstreth^[9](#_ENREF_9)^ | 1998 | USA | CS^Φ^ | ≥ 40.1 pack-years | 3660 | 1.22 | 0.93 – 1.61 |
| Longstreth^[10](#_ENREF_10)^ | 2002 | USA | CS* | Smoking | 1433 | NR | NS |
| Saji^[11](#_ENREF_11)^ | 2012 | Japan | RHS | Smoking | 220 | 2.20 | 0.91 – 5.21 |
| Saji^[12](#_ENREF_12)^ | 2012 | Japan | RHS | Smoking | 240 | 0.71 | 0.22 – 2.07 |
| Takashima[^13^](#_ENREF_13) | 2010 | Japan | CS | Smoking | 680 | 2.30 | 1.16 – 4.57 |
| Vermeer[^14^](#_ENREF_14) | 2002 | Netherlands | CS | ≥ 20 pack years | 1077 | 1.0 | 0.6 – 1.5 |
| Vermeer[^14^](#_ENREF_14) | 2002 | Netherlands | CS | >0 and <20 pack years | 1077 | 1.2 | 0.8 – 1.8 |
| Vermeer[^15^](#_ENREF_15) | 2003 | Netherlands | CS* | Current smoking habit | 668 | 1.4 | 0.6 – 3.3 |
| **ALCOHOL** | | | | | | | |
| Aono^[1](#_ENREF_1)^ | 2007 | Japan | CS | Ever drinkers | 958 | 1.19 | 0.80 – 1.79 |
| Bokura^[3](#_ENREF_3)^ | 2008 | Japan | RHS | Alcohol habit | 1151 | 1.00 | 0.98 – 1.02 |
| Fukuda[^5^](#_ENREF_5) | 2013 | Japan | CS | >1 standard drink / week | 715 | 2.11 | 1.24 – 3.58 |
| Fukuda[^16^](#_ENREF_16) | 2009 | Japan | CS | Mild consumption | 385 | 4.1 | 1.7 – 10 |
| Fukuda[^16^](#_ENREF_16) | 2009 | Japan | CS | Moderate consumption | 385 | 3.1 | 1.3 – 7.0 |
| Howard[^7^](#_ENREF_7) | 1998 | USA | CS | Past | 1737 | 1.37 | 0.90 – 2.09 |
| Howard[^7^](#_ENREF_7) | 1998 | USA | CS | Current | 1737 | 0.91 | 0.60 – 2.09 |
| Lee[^8^](#_ENREF_8) | 2000 | Korea | RHS | Mild consumption | 994 | 0.31 | 0.12 – 0.78 |
| Mukamal^[17](#_ENREF_17)^ | 2001 | USA | CS | Past | 3376 | 1.0 | 0.65 – 1.38 |
| Mukamal^[17](#_ENREF_17)^ | 2001 | USA | CS | <1standard drink / week | 3376 | 0.98 | 0.74 – 1.29 |
| Mukamal^[17](#_ENREF_17)^ | 2001 | USA | CS | 1 to <7 standard drinks / week | 3376 | 0.63 | 0.46 – 0.86 |
| Mukamal^[17](#_ENREF_17)^ | 2001 | USA | CS | 7 to <15 standard drinks / week | 3376 | 0.80 | 0.55 – 1.17 |
| Mukamal^[17](#_ENREF_17)^ | 2001 | USA | CS | ≥15 standard drinks / week | 3376 | 0.57 | 0.32-1.0 |
| Takashima[^13^](#_ENREF_13) | 2010 | Japan | CS | Alcohol habit (yes) | 680 | 2.5 | 1.39 – 4.47 |

* Longtitudinal study; RHS = Routine Health Screen; CS = Community Survey; NR = Not Reported; NS = Not Significant
