# Supplementary material for: The epidemiology of silent brain infarction: a systematic review of population-based cohorts
Source: BMC Med. 2014 Jul 9;12:119. doi: 10.1186/s12916-014-0119-0 (PMC4226994; doi:10.1186/s12916-014-0119-0)
Supplement: Additional file 5: Table S4. — Non-cardiovascular Disease risk factors for prevalent Silent Brain Infarction. [file s12916-014-0119-0-S5.docx]

**Supplementary Table 4:** Non-cardiovascular Disease risk factors for prevalent Silent Brain Infarction

| **Study** | **Year** | | **Country** | **Design** | | **Measure** | **Size** | **OR** | | **95% CI** |
| --- | --- | --- | --- | --- | --- | --- | --- | --- | --- | --- |
| **DIABETES MELLITUS OR IMPAIRED FASTING GLUCOSE** | | | | | | | | | | |
| Asumi^[2](#_ENREF_2)^ | 2010 | | Japan | RHS | | Diabetes (dichotomous) | 324 | | 0.38 | 0.05 – 2.60 |
| Bokura^[3](#_ENREF_3)^ | 2008 | | Japan | RHS | | IFG | 1151 | | 1.86 | 1.29 – 2.67 |
| Chou[^18^](#_ENREF_18) | 2011 | | Taiwan | CS/RHS | | Diabetes | 1312 | | 1.83 | 0.98-3.32 |
| Das[^4^](#_ENREF_4) | 2008 | | USA | CS | | Diabetes | 2040 | | 1.4 | 0.90 – 2.17 |
| Fukuda[^5^](#_ENREF_5) | 2013 | | Japan | CS | | Diabetes | 715 | | 3.26 | 1.09 – 9.77 |
| Heo^[6](#_ENREF_6)^ | 2010 | | Korea | RHS | | Diabetes | 1577 | | 1.63 | 0.80 – 3.29 |
| Heo^[6](#_ENREF_6)^ | 2010 | | Korea | RHS | | IFG | 1577 | | 1.01 | 0.99 – 1.02 |
| Heo^[6](#_ENREF_6)^ | 2010 | | Korea | RHS | | HbA1C | 1577 | | 0.96 | 0.66 – 1.39 |
| Howard[^7^](#_ENREF_7) | 2000 | | USA | CS | | Diabetes | 1737 | | 1.36 | 0.88 – 2.09 |
| Kwon[^19^](#_ENREF_19) | 2006 | | Korea | RHS | | IFG | 1588 | | 1.74 | 1.08 – 2.80 |
| Kwon[^20^](#_ENREF_20) | 2009 | | Korea | RHS | | IFG | 1254 | | 1.35 | 0.96 – 1.91 |
| Lee[^8^](#_ENREF_8) | 2000 | | Korea | RHS | | Diabetes | 994 | | 1.89 | 0.51 – 7.00 |
| Longstreth^[21](#_ENREF_21)^ | 1998 | | USA | CS^Φ^ | | Diabetes | 3660 | | 1.1 | 0.79 – 1.53 |
| Park[^23^](#_ENREF_23) | 2008 | | Japan | CS | | IFG | 2076 | | 2.68 | 1.77 – 4.05 |
| Saji^[11](#_ENREF_11)^ | 2012 | | Japan | RHS | | Diabetes | 220 | | 1.22 | 0.59 – 2.45 |
| Saji^[12](#_ENREF_12)^ | 2012 | | Japan | RHS | | Diabetes | 240 | | 1.86 | 0.84 – 4.08 |
| Vermeer[^14^](#_ENREF_14) | 2002 | | Netherlands | CS | | Diabetes | 1077 | | 0.7 | 0.4 – 1.5 |
| Vermeer[^15^](#_ENREF_15) | 2003 | | Netherlands | CS* | | Diabetes | 668 | | 2.9 | 1.00 – 8.5 |
| Yi[^24^](#_ENREF_24) | 2011 | | China | RHS | | Diabetes | 1008 | | 1.57 | 1.25 – 1.99 |
| **CHRONIC KIDNEY DISEASE** | | | | | | | | | | |
| Chou[^18^](#_ENREF_18) | 2011 | | Taiwan | CS/RHS | | eGFR (45.0-59.9) | 1312 | | 1.85 | 0.53-6.46 |
| Chou[^18^](#_ENREF_18) | 2011 | | Taiwan | CS/RHS | | eGFR (30-44.9) | 1312 | | 10.56 | 3.00-37.10 |
| Heo^[6](#_ENREF_6)^ | 2010 | | Korea | RHS | | Serum creatinine | 1577 | | 1.33 | 0.77 – 2.30 |
| Longstreth^[9](#_ENREF_9)^ | 1998 | | USA | CS^Φ^ | | Serum creatinine ≥1.3 vs. ≤0.9 |  | | 1.53 | 1.13 – 2.07 |
| Longstreth^[10](#_ENREF_10)^ | 2002 | | USA | CS* | | Serum creatinine >112 vs. <75 | 1433 | | 1.5 | 1.0 - 2.4 |
| Seliger^[31](#_ENREF_31)^ | 2005 | | USA | CS | | serum creatinine | 2784 | | 1.08 | 0.98 – 1.19 |
| Seliger^[31](#_ENREF_31)^ | 2005 | | USA | CS | | cystatin C | 2784 | | 1.20 | 1.09 - 1.32 |
| **METABOLIC SYNDROME** | | | | | | | | | | |
| Bokura^[3](#_ENREF_3)^ | 2008 | | Japan | RHS | | Met. Syndrome (no. of syndrome components 4 vs. 0) | 1151 | | 4.71 | 2.11-10.50 |
| Bokura^[3](#_ENREF_3)^ | 2008 | | Japan | RHS | | Met. Syndrome (dichotomous) | 1151 | | 2.43 | 1.53 – 3.87 |
|  |  | |  |  | |  |  | |  |  |
| Kwon[^19^](#_ENREF_19) | 2006 | | Korea | RHS | | Met. Syndrome | 1588 | | 2.18 | 1.38 - 3.44 |
| Kwon[^20^](#_ENREF_20) | 2009 | | Korea | RHS | | Met. Syndrome | 1254 | | 1.68 | 1.15 – 2.44 |
| Park[^23^](#_ENREF_23) | 2008 | | Japan | CS | | Met. Syndrome | 2076 | | 6.52 | 4.30 - 9.90 |
| **OBESITY** | | | | | | | | | | |
| Aono^[1](#_ENREF_1)^ | | 2007 | Japan | | CS | BMI ≥ 25 vs. <25 kg/m^2^ | 958 | 0.72 | | 0.53 – 0.97 |
| Asumi^[2](#_ENREF_2)^ | | 2010 | Japan | | RHS | BMI > 25 vs. <25 kg/m^2^ | 324 | 1.12 | | 0.32 – 3.91 |
| Bokura^[3](#_ENREF_3)^ | | 2008 | Japan | | RHS | BMI ≥ 25 kg/m^2^ | 1151 | 1.55 | | 1.05 – 2.27 |
| Howard[^7^](#_ENREF_7) | | 1998 | USA | | CS | BMI/SD | 1737 | 0.90 | | 0.75 – 1.08 |
| Kwon[^19^](#_ENREF_19) | | 2006 | Korea | | RHS | Large WC | 1588 | 0.70 | | 0.41 – 1.18 |
| Kwon[^20^](#_ENREF_20) | | 2009 | Korea | | RHS | Large WC | 1254 | 1.23 | | 0.72 – 2.09 |
| Lee[^8^](#_ENREF_8) | | 2000 | Korea | | RHS | Obesity | 994 | 1.00 | | 0.49 – 2.03 |
| Park[^23^](#_ENREF_23) | | 2008 | Japan | | CS | Large WC (dichotomised) | 2076 | 8.35 | | 5.59 – 12.5 |
| Park[^23^](#_ENREF_23) | | 2008 | Japan | | CS | Large WC (highest vs. lowest tertile) | 2076 | 4.30 | | 2.40 – 7.71 |

^Φ^ Silent lacunes only assessed (i.e. excludes silent cortical infarcts); * Longtitudinal study; CS = Community Survey; RHS = Routine Health Screen; IFG = Impaired Fasting Glucose; eGFR = estimated Glomerular Filtration Ratio; Met. Syndrome = Metabolic Syndrome; BMI = Body Mass Index; SD = Standard Deviation; WC = Waist Circumference.
