# Supplementary material for: The epidemiology of silent brain infarction: a systematic review of population-based cohorts
Source: BMC Med. 2014 Jul 9;12:119. doi: 10.1186/s12916-014-0119-0 (PMC4226994; doi:10.1186/s12916-014-0119-0)
Supplement: Additional file 6: Table S5. — Laboratory indicators as risk indicators of prevalent Silent Brain Infarction. [file s12916-014-0119-0-S6.docx]

**Supplementary Table 5:** Laboratory indicators as risk indicators of prevalent Silent Brain Infarction

| **Authors** | **Year** | **Country** | **Design** | **Disease marker** | **Sample** | **OR** | **95% CI** |
| --- | --- | --- | --- | --- | --- | --- | --- |
| **DYSLIPIDEMIA** | | | | | | | |
| Aono^[1](#_ENREF_1)^ | 2007 | Japan | CS | Hypercholesterolemia | 958 | 1.20 | 0.89 – 1.61 |
| Asumi^[2](#_ENREF_2)^ | 2010 | Japan | RHS | Total cholesterol per mmol/L | 324 | 3.75 | 1.45-9.68 |
| Asumi^[2](#_ENREF_2)^ | 2010 | Japan | RHS | LDL per mmol/L | 324 | 2.54 | 1.03-6.27 |
| Asumi^[2](#_ENREF_2)^ | 2010 | Japan | RHS | HDL per mmol/L | 324 | 4.52 | 0.97 – 21.0 |
| Asumi^[2](#_ENREF_2)^ | 2010 | Japan | RHS | Triglycerides | 324 | 1.36 | 0.48 – 3.84 |
| Bokura^[3](#_ENREF_3)^ | 2008 | Japan | RHS | Dyslipidemia | 1151 | 1.24 | 0.85 – 1.81 |
| Das[^4^](#_ENREF_4) | 2008 | USA | CS | Total cholesterol | 2040 | 1.10 | 0.95 – 1.27 |
| Das[^4^](#_ENREF_4) | 2008 | USA | CS | HDL | 2040 | 0.97 | 0.82 – 1.13 |
| Heo^[6](#_ENREF_6)^ | 2010 | Korea | RHS | Dyslipidemia | 1577 | 0.91 | 0.50 – 1.65 |
| Howard[^7^](#_ENREF_7) | 2000 | USA | CS | Triglycerides/SD | 1737 | 0.97 | 0.80 – 1.18 |
| Howard[^7^](#_ENREF_7) | 2000 | USA | CS | HDL/SD | 1737 | 1.02 | 0.84 – 1.25 |
| Kwon[^19^](#_ENREF_19) | 2006 | Korea | RHS | High triglycerides | 1588 | 1.54 | 0.91 – 2.61 |
| Kwon[^19^](#_ENREF_19) | 2006 | Korea | RHS | Low HDL | 1588 | 1.08 | 0.64 – 1.82 |
| Kwon[^20^](#_ENREF_20) | 2009 | Korea | RHS | High Triglycerides | 1254 | 1.39 | 0.89 – 2.17 |
| Kwon[^20^](#_ENREF_20) | 2009 | Korea | RHS | Low HDL | 1254 | 0.84 | 0.58 – 1.22 |
|  |  |  |  |  |  |  |  |
| Lee[^8^](#_ENREF_8) | 2000 | Korea | RHS | Total cholesterol | 994 | 1.07 | 0.45 – 2.56 |
| Longstreth^[21](#_ENREF_21)^ | 1998 | USA | CS^Φ^ | LDL ≥ 144 vs. ≤100 | 3660 | 1.13 | 0.87 – 1.47 |
| Park[^23^](#_ENREF_23) | 2008 | Japan | CS | High triglycerides | 2076 | 2.82 | 1.83 – 4.33 |
| Park[^23^](#_ENREF_23) | 2008 | Japan | CS | Low HDL | 2076 | 0.71 | 0.34 – 1.47 |
| Saji^[11](#_ENREF_11)^ | 2012 | Japan | RHS | Hypercholesterolemia | 220 | 1.05 | 0.57 – 1.94 |
| Saji^[12](#_ENREF_12)^ | 2012 | Japan | RHS | Hypercholesterolemia | 240 | 1.31 | 0.61 – 2.86 |
| Vermeer[^15^](#_ENREF_15) | 2003 | Netherlands | CS* | Total cholesterol / SD | 668 | 1.08 | 0.76 – 1.53 |
| Vermeer[^15^](#_ENREF_15) | 2003 | Netherlands | CS* | HDL / SD | 668 | 0.81 | 0.56 – 1.15 |
| Yi[^24^](#_ENREF_24) | 2011 | China | RHS | LDL / SD | 1008 | 1.22 | 0.99 – 1.51 |
| Yi[^24^](#_ENREF_24) | 2011 | China | RHS | Lipoprotein α (HDL)/ SD | 1008 | 1.13 | 1.03 – 1.23 |
| **TOTAL PLASMA HOMOCYSTEINE** | | | | | | | |
| Das[^4^](#_ENREF_4) | 2008 | USA | CS | tHcy mmol/L | 2040 | 2.23 | 1.42 – 3.51 |
| Longstreth^[32](#_ENREF_32)^ | 2004 | USA | CS | tHcy (5^th^ vs. 1^st^ quintile) | 622 | 0.85 | 0.47 – 1.52 |
| Longstreth^[32](#_ENREF_32)^ | 2004 | USA | CS* | tHcy (5^th^ vs. 1^st^ quintile) | 223 | 1.00 | 0.27 – 3.69 |
| Vermeer[^33^](#_ENREF_33) | 2002 | Netherlands | CS | tHcy / SD | 1077 | 1.24 | 1.06 – 1.45 |
| Seshadri | 2008 | USA | CS | tHcy (Q4 vs. Q1-3) | 1663 | 1.61 | 1.14 – 2.25 |
| Vermeer[^15^](#_ENREF_15) | 2003 | Netherlands | CS* | tHcy | 668 | 1.31 | 0.95 – 1.82 |
| **COAGULATION FACTORS** | | | | | | | |
| Aono^[1](#_ENREF_1)^ | 2007 | Japan | CS | fibrinogen (per 1 SD) | 958 | 1.26 | 1.09 – 1.46 |
| Knuiman^[34](#_ENREF_34)^ | 2001 | USA | CS | fibrinogen (per 1 SD) | 1393 | 1.21 | 1.02 – 1.44 |
| Knuiman^[34](#_ENREF_34)^ | 2001 | USA | CS | Factor VII | 1393 | 0.98 | 0.72 – 1.08 |
| Knuiman^[34](#_ENREF_34)^ | 2001 | USA | CS | Factor VIII | 1393 | 1.07 | 0.72 – 1.08 |
| Knuiman^[34](#_ENREF_34)^ | 2001 | USA | CS | Antithrombin III | 1393 | 0.93 | 0.77 – 1.11 |
| Knuiman^[34](#_ENREF_34)^ | 2001 | USA | CS | aPTT | 1393 | 1.04 | 0.87 – 1.25 |
| Knuiman^[34](#_ENREF_34)^ | 2001 | USA | CS | Platelet count | 1393 | 1.05 | 0.87 – 1.26 |
| Knuiman^[34](#_ENREF_34)^ | 2001 | USA | CS | Protein C | 1393 | 0.77 | 0.62 – 0.97 |
| Gottesman^[35](#_ENREF_35)^ | 2009 | USA | Case-control | D-dimer | 410 | 1.76 | 1.02 – 3.0 |
| Gottesman^[35](#_ENREF_35)^ | 2009 | USA | Case-control | von Willebrand factor | 410 | 2.0 | 1.2 – 3.6 |
| Knuiman^[34](#_ENREF_34)^ | 2001 | USA | CS | von Willebrand factor | 1881 | 1.15 | 0.97 – 1.37 |
| **INFLAMMATORY MARKERS** | | | | | | | |
| Heo^[6](#_ENREF_6)^ | 2010 | Korea | RHS | hs-CRP | 1577 | 0.94 | 0.59 – 1.51 |
| Hoshi[^36^](#_ENREF_36) | 2005 | Japan | RSH | hs-CRP | 194 | 1.50 | 1.00 – 2.24 |
| Kwon[^19^](#_ENREF_19) | 2006 | Korea | RHS | hs-CRP | 1588 | 1.07 | 0.92 – 1.23 |
| Satizabal^[37](#_ENREF_37)^ | 2012 | France | CS | hs-CRP | 1841 | NR | NS |
| Satizabal^[37](#_ENREF_37)^ | 2012 | France | CS | IL-6 | 1841 | NR | NS |
| **HYPERURICEMIA** | | | | | | | |
| Asumi^[2](#_ENREF_2)^ | 2010 | Japan | RHS | Hyperuricemia (dichotomous) | 324 | 2.66 | 0.80 – 8.88 |
| Heo^[38](#_ENREF_38)^ | 2010 | Korea | RHS | serum uric acid (highest vs. lowest quartile) | 1577 | 1.79 | 1.11 – 2.91 |
| Yi[^24^](#_ENREF_24) | 2011 | China | RHS | Uric acid | 1008 | 1.002 | 1.000 – 1.003 |
| **CARDIOVASCULAR BIOMARKERS** | | | | | | | |
| Dadu^[39](#_ENREF_39)^ | 2013 | USA | CS | NT-proBNP (4^th^ vs. 1^st^ quartile) | 1501 | 3.50 | 2.03 – 6.20 |
| Dadu^[39](#_ENREF_39)^ | 2013 | USA | CS* | NT-proBNP (4^th^ vs. 1^st^ quartile) | 891 | 2.18 | 1.38 – 3.47 |
| Dadu^[39](#_ENREF_39)^ | 2013 | USA | CS | hs-cTnT (5^th^ vs. 1^st^ quintile) | 1502 | 3.03 | 1.57 – 5.82 |
| Dadu^[39](#_ENREF_39)^ | 2013 | USA | CS* | hs-cTnT (4^th^ vs. 1^st^ quintile) | 892 | 2.31 | 1.40 – 3.81 |
| Dadu^[39](#_ENREF_39)^ | 2013 | USA | CS* | hs-cTnT (5^th^ vs. 1^st^ quintile) | 892 | 1.63 | 0.78 – 3.31 |
| Pikula^[40](#_ENREF_40)^ | 2009 | USA | CS | ADMA (per SD) | 2013 | 1.16 | 1.01 – 1.33 |

^Φ^Silent Lacunar Infarction; *Longtitudinal Study; SD = Standard Deviation; LDL = Low Density Lipoprotein; HDL = High Density Lipoprotein; tHcy = total homocysteine; Q4 = 4^th^ Quartile; Q1 = 1^st^ Quartile; hs-CRP = High sensitivity C-reactive protein; IL-6 = Interleukin-6; BNP = Brain Natriuretic Peptide; TnT = Troponin T; ADMA =
