# Supplementary material for: The epidemiology of silent brain infarction: a systematic review of population-based cohorts
Source: BMC Med. 2014 Jul 9;12:119. doi: 10.1186/s12916-014-0119-0 (PMC4226994; doi:10.1186/s12916-014-0119-0)
Supplement: Additional file 7: Table S6. — Cardiovascular disease as a risk factor for prevalent Silent Brain Infarct. [file s12916-014-0119-0-S7.docx]

**Supplementary Table 6:** Cardiovascular disease as a risk factor for prevalent Silent Brain Infarct

| **Study** | | **Year** | **Country** | | | **Design** | | | **Measure** | **Size** | | | | **OR** | | | | **95% CI** | |
| --- | --- | --- | --- | --- | --- | --- | --- | --- | --- | --- | --- | --- | --- | --- | --- | --- | --- | --- | --- |
| **HYPERTENSION (HTN)** | | | | | | | | | | | | | | | | | | | |
| Aono^[1](#_ENREF_1)^ | | 2007 | Japan | | | CS | | | 24-hour SBP / SD increase | 958 | | | | 1.23 | | | | 1.10 – 1.39 | |
| Aono^[1](#_ENREF_1)^ | | 2007 | Japan | | | CS | | | Treated HTN | 958 | | | | 2.02 | | | | 1.49 – 2.75 | |
| Asumi^[2](#_ENREF_2)^ | | 2010 | Japan | | | RHS | | | HTN (dichotomous) | 324 | | | | 3.98 | | | | 1.19 – 13.3 | |
| Bokura^[3](#_ENREF_3)^ | | 2008 | Japan | | | RHS | | | HTN | 1151 | | | | 1.54 | | | | 1.08 – 2.20 | |
| Chou[^18^](#_ENREF_18) | | 2011 | Taiwan | | | CS/RHS | | | HTN | 1312 | | | | 2.56 | | | | 1.47-4.47 | |
| Das[^4^](#_ENREF_4) | | 2008 | USA | | | CS | | | HTN | 2040 | | | | 1.56 | | | | 1.15 – 2.11 | |
| Fukuda[^5^](#_ENREF_5) | | 2013 | Japan | | | CS | | | HTN | 715 | | | | 4.04 | | | | 2.41 – 6.77 | |
| Heo^[6](#_ENREF_6)^ | | 2010 | Korea | | | RHS | | | HTN | 1577 | | | | 2.58 | | | | 1.26 – 5.30 | |
| Howard[^7^](#_ENREF_7) | | 2000 | USA | | | CS | | | HTN | 1737 | | | | 2.00 | | | | 1.41 – 2.86 | |
| Kwon[^19^](#_ENREF_19) | | 2006 | Korea | | | RHS | | | HTN | 1588 | | | | 3.75 | | | | 2.05 – 6.85 | |
| Kwon[^20^](#_ENREF_20) | | 2009 | Korea | | | RHS | | | HTN | 1254 | | | | 1.89 | | | | 1.23 – 2.91 | |
| Lee[^8^](#_ENREF_8) | | 2000 | Korea | | | RHS | | | HTN | 994 | | | | 3.47 | | | | 1.71 – 7.03 | |
| Longstreth^[21](#_ENREF_21)^ | | 1998 | USA | | | CS^Φ^ | | | DBP ≥ 79 vs. ≤ 64 | 3660 | | | | 1.57 | | | | 1.21 – 2.05 | |
| Matsumoto[^22^](#_ENREF_22) | | 2007 | Japan | | | RHS | | | Treated HTN | 476 | | | | 2.22 | | | | 1.11-4 .43 | |
| Park[^23^](#_ENREF_23) | | 2008 | Japan | | | CS | | | HTN | 2076 | | | | 2.94 | | | | 1.90 – 4.56 | |
| Saji^[11](#_ENREF_11)^ | | 2012 | Japan | | | RHS | | | HTN | 220 | | | | 3.87 | | | | 1.58 – 11.7 | |
| Saji^[12](#_ENREF_12)^ | | 2012 | Japan | | | RHS | | | HTN | 240 | | | | 1.60 | | | | 0.62 – 4.70 | |
| Takashima[^13^](#_ENREF_13) | | 2010 | Japan | | | CS | | | HTN | 680 | | | | 3.47 | | | | 1.99 – 6.03 | |
| Vermeer[^14^](#_ENREF_14) | | 2002 | Netherlands | | | CS | | | HTN | 1077 | | | | 2.3 | | | | 1.6 – 3.2 | |
| Vermeer[^14^](#_ENREF_14) | | 2002 | Netherlands | | | CS | | | SBP/ SD increase | 1077 | | | | 1.45 | | | | 1.23 – 1.71 | |
| Vermeer[^14^](#_ENREF_14) | | 2002 | Netherlands | | | CS | | | DBP / SD increase | 1077 | | | | 1.27 | | | | 1.08 – 1.49 | |
| Vermeer[^14^](#_ENREF_14) | | 2002 | Netherlands | | | CS | | | Pulse Pressure | 1077 | | | | 1.34 | | | | 1.13 – 1.58 | |
| Vermeer[^15^](#_ENREF_15) | | 2003 | Netherlands | | | CS* | | | HTN | 1077 | | | | 1.2 | | | | 0.6 – 2.4 | |
| Yi[^24^](#_ENREF_24) | | 2011 | China | | | RHS | | | HTN | 1008 | | | | 1.36 | | | | 1.17 – 1.58 | |
| **PRIOR STROKE OR TIA** | | | | | | | | | | | | | | | | | | | |
| Price[^25^](#_ENREF_25) | | 1997 | USA | | | CS | Prior stroke | | | | 3647 | | | | | 4.12 | | | 2.96 – 5.72 |
| **CAROTID ARTERY DISEASE** | | | | | | | | | | | | | | | | | | | |
| Asumi^[2](#_ENREF_2)^ | | 2010 | Japan | | | RHS | IMT (highest vs. lowest tertile) | | | 324 | | | | | 5.51 | | | | 1.31 – 23.1 |
| Chou[^18^](#_ENREF_18) | | 2011 | Taiwan | | | CS/RHS | Carotid plaque score ≥4 | | | 1312 | | | | | 3.08 | | | | 1.64-5.78 |
| Das[^4^](#_ENREF_4) | | 2008 | USA | | | CS | ≥ 25% stenosis | | | 2040 | | | | | 1.62 | | | | 1.13 – 2.34 |
| Das[^4^](#_ENREF_4) | | 2008 | USA | | | CS | CCA IMT | | | 2040 | | | | | 1.20 | | | | 1.02 – 1.40 |
| Das[^4^](#_ENREF_4) | | 2008 | USA | | | CS | ICA IMT | | | 2040 | | | | | 1.32 | | | | 1.13 – 1.54 |
| Longstreth^[21](#_ENREF_21)^ | | 1998 | USA | | | CS^Φ^ | ≥ 50% stenosis | | | 3660 | | | | | 1.86 | | | | 1.23 – 2.81 |
| Manolio^[26](#_ENREF_26)^ | | 1999 | USA | | | CS | 50-74% stenosis | | | 3502 | | | | | 1.81 | | | | 1.16 – 2.84 |
| Manolio^[26](#_ENREF_26)^ | | 1999 | USA | | | CS | ≥75% stenosis | | | 3502 | | | | | 2.43 | | | | 1.32 – 4.48 |
| Manolio^[26](#_ENREF_26)^ | | 1999 | USA | | | CS | Calcified density | | | 3502 | | | | | 1.73 | | | | 1.03 – 2.90 |
| Matsumoto[^22^](#_ENREF_22) | | 2007 | Japan | | | RHS | Carotid plaque | | | 476 | | | | | 2.69 | | | | 1.59 – 4.56 |
| Matsumoto[^22^](#_ENREF_22) | | 2007 | Japan | | | RHS | Intima-media thickness | | | 476 | | | | | 2.4 | | | | 1.02 – 5.65 |
| Romero[^27^](#_ENREF_27) | | 2009 | USA | | | CS | ≥50% stenosis | | | 1971 | | | | | 2.53 | | | | 1.17 – 5.44 |
| Romero[^27^](#_ENREF_27) | | 2009 | USA | | | CS | Log IMT | | | 1971 | | | | | 1.21 | | | | 1.03 – 1.43 |
| Saji^[11](#_ENREF_11)^ | | 2012 | Japan | | | RHS | IMT | | | 220 | | | | | 1.96 | | | | 1.33 – 2.93 |
| Saji^[12](#_ENREF_12)^ | | 2012 | Japan | | | RHS | IMT | | | 240 | | | | | 1.30 | | | | 0.82 – 2.07 |
| Vermeer[^15^](#_ENREF_15) | | 2003 | Netherlands | | | CS* | IMT / SD | | | 668 | | | | | 1.31 | | | | 1.01 – 1.71 |
| Vermeer[^15^](#_ENREF_15) | | 2003 | Netherlands | | | CS* | Carotid artery plaques | | | 668 | | | | | 1.4 | | | | 0.85 – 1.68 |
| Yi[^24^](#_ENREF_24) | | 2011 | China | | | RHS | IMT | | | 1008 | | | | | 1.54 | | | | 1.09 – 2.16 |
| **CORONARY ARTERY DISEASE (CAD)** | | | | | | | | | | | | | | | | | | | |
| Asumi^[2](#_ENREF_2)^ | | 2010 | Japan | | | RHS | | CAD (dichotomous) | | | | 324 | | | | | 3.46 | | 0.38 – 31.5 |
| Heo^[6](#_ENREF_6)^ | | 2010 | Korea | | | RHS | | CAD | | | | 1577 | | | | | 2.53 | | 1.18 – 5.40 |
| Kwon[^19^](#_ENREF_19) | | 2006 | Korea | | | RHS | | CAD | | | | 1588 | | | | | 2.83 | | 1.38 – 5.82 |
| Lee[^8^](#_ENREF_8) | | 2000 | Korea | | | RHS | | CAD | | | | 994 | | | | | 2.22 | | 0.53 – 9.34 |
| Longstreth^[21](#_ENREF_21)^ | | 1998 | USA | | | CS | | CAD^Φ^ | | | | 3660 | | | | | 1.34 | | 1.08 – 1.68 |
| Saji^[11](#_ENREF_11)^ | | 2012 | Japan | | | RHS | | CAD | | | | 220 | | | | | 2.64 | | 1.30 – 5.4 |
| Saji^[12](#_ENREF_12)^ | | 2012 | Japan | | | RHS | | CAD | | | | 240 | | | | | 1.79 | | 0.74 – 4.17 |
| **ATRIAL FIBRILLATION (AF)** | | | | | | | | | | | | | | | | | | | |
| Aono^[1](#_ENREF_1)^ | | 2007 | Japan | | | CS | | AF | | | | 958 | | | | | 1.06 | | 0.44 – 2.52 |
| Das[^4^](#_ENREF_4) | | 2008 | USA | | | CS | | AF | | | | 2040 | | | | | 2.16 | | 1.07 – 4.40 |
| Price[^25^](#_ENREF_25) | | 1997 | USA | | | CS | | AF | | | | 3647 | | | | | NR | | NS |
| **HEART FAILURE** | | | | | | | | | | | | | | | | | | | |
| Das[^28^](#_ENREF_28) | 2008 | | | USA | CS | | | | LVH | | | | 2040 | | | | 1.51 | | 0.17 – 13.04 |
| Lee[^29^](#_ENREF_29) | 2000 | | | Korea | RHS | | | | Cardiomegaly on CXR | | | | 994 | | | | 2.52 | | 0.64 – 9.95 |
| Russo[^30^](#_ENREF_30) | 2013 | | | USA | CS | | | | LAVmax | | | | 455 | | | | 1.15 | | 0.88 – 1.50 |
| Russo[^30^](#_ENREF_30) | 2013 | | | USA | CS | | | | LAVmin | | | | 455 | | | | 1.37 | | 1.04 – 1.80 |
| Russo[^30^](#_ENREF_30) | 2013 | | | USA | CS | | | | LAEV | | | | 455 | | | | 0.77 | | 0.58 – 1.02 |
| Russo[^30^](#_ENREF_30) | 2013 | | | USA | CS | | | | LAEF | | | | 455 | | | | 1.49 | | 1.11 – 2.0 |

^Φ^ Silent lacunes only assessed (i.e. excludes silent cortical infarcts); * Longtitudinal study; CS = Community Survey; RHS = Routine Health Screen; SBP = Systolic Blood Pressure; SD = Standard Deviation; HTN = Hypertension; DBP = Diastolic Blood Pressure; IMT = Intima Media Thickness; CCA = Common Carotid Artery; ICA = Internal Carotid Artery; LVH = Left Ventricular Hypertrophy; LAV = Left Atrial Volume; LAEV = Left Atrial Ejection Volume; LAEF = Left Atrial Ejection Fraction.
