# Supplementary material for: The epidemiology of silent brain infarction: a systematic review of population-based cohorts
Source: BMC Med. 2014 Jul 9;12:119. doi: 10.1186/s12916-014-0119-0 (PMC4226994; doi:10.1186/s12916-014-0119-0)
Supplement: Additional file 8: Figure S2a. — Mean prevalence of silent brain infarction, by mean age, differentiated by origin of study. [file s12916-014-0119-0-S8.docx]

**Supplementary Figure 2a:** Mean prevalence of silent brain infarction, by mean age, differentiated by origin of study

**Figure 2b:** Mean prevalence of silent brain infarction, by mean age, differentiated by origin of study, only including community surveys and excluding one outlier study (Aono et al., 2007)
